# European Brown hare (*Lepus europaeus*) as a source of emerging and re‐emerging pathogens of Public Health importance: A review

**DOI:** 10.1002/vms3.248

**Published:** 2020-02-23

**Authors:** Constantina N. Tsokana, Christos Sokos, Alexios Giannakopoulos, Periklis Birtsas, George Valiakos, Vassiliki Spyrou, Labrini V. Athanasiou, Angeliki Rodi Burriel, Charalambos Billinis

**Affiliations:** ^1^ Department of Microbiology and Parasitology Faculty of Veterinary Medicine University of Thessaly Karditsa Greece; ^2^ Research Division Hunting Federation of Macedonia and Thrace Thessaloniki Greece; ^3^ Department of Forestry and Natural Environment Administration Technological Institute of Thessaly Karditsa Greece; ^4^ Department of Animal Production Technological Education Institute of Thessaly Larissa Greece; ^5^ Department of Medicine Faculty of Veterinary Medicine University of Thessaly Karditsa Greece; ^6^ Department of Nursing University of Peloponnesus Sparti Greece

**Keywords:** *Brucella*, Crimean–Congo haemorrhagic fever virus, *Francisella tularensis*, Hepatitis E virus, *Leishmania* spp. *Lepus europaeus*, *Toxoplasma gondii*, *Yersinia* spp.

## Abstract

European brown hare (*Lepus europaeus,* EBH) is probably the most important game animal in Europe throughout its historical distribution. The decline in its populations across its geographic range in Europe have been attributed to factors such as reproductive rate and the ability for adaptation, climate, feed availability, predators, anthropogenic factors and diseases. Apart from common diseases of hares with a high impact on their mortality such as European Brown hare Syndrome, EBH has been involved in the epidemiology of pathogens with zoonotic potential. In this work, the role of EBH as a source of Crimean–Congo haemorrhagic fever virus (CCHFV), Hepatitis E virus (HEV), *Yersinia* spp., *Brucella* spp., *Francisella tularensis*, *Toxoplasma gondii* and *Leishmania infantum* is discussed. Hares may significantly contribute to the epidemiology of important emerging zoonotic pathogens through maintenance of high endemicity levels as in the case of CCHFV, as a reservoir of important pathogens such as *Yersinia* spp., *B. suis*, *F. tularensis* and *L. infantum* and as a potential source of *T. gondii* for other animals, especially for carnivores but also for humans. However, EBH may also be a host of minor importance as in the case of HEV. The continuous surveillance of hare populations will enable the collection of information on the population health status and the pathogens currently circulating in the area posing risk for wildlife, domestic animals and humans. The possible live animal translocations of infected hares, the fact that this species acts as a host of vectors (fleas, ticks, mosquitoes and sandflies) and the prey of carnivores and omnivores that travel in great distances getting into contact with domestic animals and humans, further highlights the need to be included in surveillance studies. Besides, the hunter‐harvested EBH population is an excellent indicator for recent pathogen transmission due to its short lifespan.

## Abstract 2

The present review provides an overview of the role of European Brown Hare as a source of Crimean–Congo haemorrhagic fever virus (CCHFV), Hepatitis E virus (HEV), *Yersinia* spp., *Brucella* spp., *Francisella tularensis*, *Toxoplasma gondii* and *Leishmania infantum* aiming to highlight the contribution of this species to the epidemiology of important emerging zoonotic pathogens.

## 1 INTRODUCTION

*Lepus europaeus* belongs to the order *Lagomorpha*, Family *Leporidae*, Genus *Lepus* with 31 other hare and jackrabbit species. *Le. europaeus* is probably the most important game animal in Europe throughout its historical distribution, including those areas where it has been introduced, being widespread and abundant across its geographic range. The current Eurasian distribution of *Le. europaeus* extends from northern Spain, to introduced populations in the United Kingdom and southern Scandinavia, south to northern portions of the Middle East, and has naturally expanded east to Siberia (IUCN, 2008). This species has been introduced as a game species extensively to countries including Argentina, Australia, Barbados, Brazil, Canada, Chile, Falkland Islands, New Zealand, the United Kingdom and the United States (Chapman et al., 1990).

Being a highly adaptable species that can persist in various habitat types, high arctic tundra, steppe, agricultural pasture, tropical savanna and desert are all types of habitats occupied by hares (Smith, Vaughan Jennings, & Harris, 2005). This species keeps the so‐called home range; individual home ranges vary from 10 to 300 ha and often overlap on favoured feeding areas. Hares usually travel up to 1.8 km in search of suitable graze. However, it has been reported that they may travel up to 15 km in one night while feeding (Chapman et al., 1990).

Decline in the populations of *Le. europaeus* has been experienced in many areas across its geographic range in Europe, beginning in the 1960s, in association with the intensification of agricultural practices (Smith et al., 2005). There is a considerable concern over recent declines in Europe, but the populations are not really threatened. Hare population densities range from 0.1/ha to 3.4/ha (IUCN, 2008). The decline in this species population has garnered its protection under the Bern Convention as an Appendix III listing (Vaughan, Lucas, Harris, & White, 2003). However, in Norway, Germany, Austria and Switzerland, population declines have resulted in country‐specific Red Listing as "near threatened" or "threatened" (IUCN, 2008).

External factors such as climate, feed availability, diseases and predators, internal factors such as reproductive rate and ability for adaptation and anthropogenic factors may affect the population density of this species (Treml, Pikula, Bandouchova, & Horakova, 2007). To address population declines, restocking programmes involved the release of captive‐bred individuals into the wild as well as the importation and release of wild hares from one country to another in several European countries. However, wildlife restocking operations created concern about the risk of pathogens introduction in new areas through translocations and the threat posed on the integrity of locally adapted genetic diversity (Spyrou et al., 2013). Apart from common diseases of hares with high impact on their mortality such as pseudotuberculosis and European Brown hare Syndrome, hare has been involved in the epidemiology of important pathogens such as *Francisella tularensis*, *Leptospira* spp., *Borrelia* spp., *Toxoplasma gondii* and *Leishmania infantum* posing threat especially for human health (Bártová, Sedlák, Treml, Holko, & Literák, 2010; Ebani et al., 2016; Hartskeerl & Terpstra, 1996; Molina et al., 2012; Tagliabue, Figarolli, & D’Incau, 2016; Tälleklint & Jaenson, 1993; Treml, Pikula, & Holešovská, 2003; Treml et al., 2007).

The present review provides an overview of the available information on selected viral, bacterial and protozoal emerging and re‐emerging pathogens of Public Health importance detected in European Brown hare. The pathogens discussed herein were selected based on the availability of data in literature regarding the hare involvement in their epidemiology. In this work, the role of the EBH as a source of Crimean–Congo haemorrhagic fever virus (CCHFV), Hepatitis E virus (HEV), *Yersinia* spp., *Brucella* spp., *F. tularensis*, *T. gondii* and *L. infantum* is discussed.

## 2 VIRAL PATHOGENS

### 2.1 Crimean–Congo haemorrhagic fever virus

Crimean–Congo haemorrhagic fever virus (CCHFV) has been described over a wide geographic area and it is endemic in focal areas in Asia, Europe and Africa. The natural vector and reservoir have been identified as *Hyalomma* spp. ticks, and the geographic distribution of human cases closely mirrors the vector distribution. The virus can be also transmitted to humans by direct contact with blood or tissues of viraemic patients or animals (Aradaib et al., 2010; Papa, Mirazimi, Köksal, Estrada‐Pena, & Feldmann, 2015). CCHFV circulates in nature in unnoticed enzootic tick–vertebrate–tick cycles (Spengler, Bergeron, & Rollin, 2016). Thus, a critical transmission rate of CCHFV is reached when adequate density of reservoir hosts is established together with the presence and abundance of the tick vectors (Estrada‐Peña, Jameson, Medlock, Vatansever, & Tishkova, 2012; Hartemink, Randolph, Davis, & Heesterbeek, 2008). Clinical disease is restricted to humans. However, asymptomatic CCHFV infection has been reported in numerous wild and domestic animals (Nalca & Whitehouse, 2007; Spengler et al., 2016). Cattle, sheep, goats, horses, pigs, dogs and chickens are among the most investigated domestic animals internationally with cattle (79.1% in Afghanistan), sheep (75% in Afghanistan) (Mustafa et al., 2011), goats (66% in Turkey) (Tuncer et al., 2014) and horses (58.8% in Iraq) (Tantawi, Shony, & Al‐Tikriti, 1981) reaching particularly high seroprevalence in some areas.

Among the wild animals investigated, considerable seroprevalence was reported in hares (3%–22%), buffalo (10%–20%) and rhinoceroses (40%–68%). Data on European brown hare come from Russia (20%) and Hungary (6%) (Hoogstraal, 1979; Németh et al., 2013). However, seropositivity has been also reported in *L. capensis* in Zimbabwe (22.6%) and Turkmenistan, *Lepus* spp. in Zimbabwe (14.3%), Bulgaria (3%) and Iran and *L. saxatilis* in Zimbabwe (14.5%) (Hoogstraal, 1979; Shepherd, Swanepoel, Shepherd, McGillivray, & Searle, 1987; Spengler et al., 2016).

Reports of CCHFV isolation from animals are limited to cattle (Kenya and Nigeria), goat (Nigeria and Senegal), hedgehog (Nigeria) and European brown hares (Crimea). This scarcity of isolates from animals together with the data obtained from experimental studies, likely reflect a relatively brief viraemic period. Thus, the difficulty in identifying infected animals is more likely due to absence or mild clinical disease (Fagbami, Tomori, Fabiyi, & Isoun, 1975; Shepherd, Swanepoel, Cornel, & Mathee, 1989; Smirnova, 1979) than to a real lack of infection in animals.

The serological studies conducted so far in different endemic regions of Europe, Africa and Asia have shown that the principal hosts of adult *Hyalomma* spp. ticks, the large herbivores, exhibit the highest seroprevalence (Nalca & Whitehouse, 2007). However, the detection of antibodies against CCHFV does not necessarily imply that the animal species examined are susceptible to infection with CCHFV or develop sufficiently high blood viral titre to efficiently infect ticks (Shepherd et al., 1989; Shepherd, Swanepoel, Shepherd, Leman, & Mathee, 1991). The latter has been shown in sheep, calves, scrub hares (*L. saxatilis*) and ostriches during experimental infection studies conducted since 1970 (Bente et al., 2013). However, these data are limited and cannot lead to solid conclusions on the role of various animal species in the maintenance of CCHFV in enzootic areas. However, it has been suggested that hares act as reservoir hosts for CCHFV in the former Union of Soviet Socialist Republics (USSR) and Bulgaria, as well as in southern Africa (Hoogstraal, 1979; Shepherd et al., 1989). This suggestion was further supported by the reported seroprevalences, the fact that the immature *Hyalomma* spp. ticks feed primarily on small vertebrates among which hares (Camicas et al., 1990) and the results of experimental infection studies that showed the ability of *L. saxatilis* to act as a proficient amplifier of the virus with viraemic transmission of virus to ticks (Shepherd et al., 1991). It has been suggested that in areas where small mammals, such as hares, and large mammals, such as sheep and cattle, are abundant and co‐exist, the virus circulation is silent with occasional human cases. On the contrary, when small mammals are abundant and few large mammals exist, the questing adult *Hyalomma* spp. will attack any humans who enter the area in an urgent need to find a large animal source for their next blood meal. Although based on old studies, and not relevant to the EBH, the above‐mentioned facts support the theory that hares may not serve as direct sources of viral transmission as viraemic livestock does, but they aid principally in maintaining high levels of CCHFV endemicity (Spengler et al., 2016).

### 2.2 Hepatitis E virus (HEV)

HEV‐1 and HEV‐2 are mainly restricted to humans and have been responsible for large waterborne epidemics of hepatitis E. However, several primate species have been also shown to be susceptible for HEV‐1 and HEV‐2 (Pavio, Meng, & Doceul, 2015; Spahr, Knauf‐Witzens, Vahlenkamp, Ulrich, & Johne, 2017). HEV‐3 and HEV‐4 have been detected in both humans and animals, and are the main cause of sporadic cases of hepatitis E in humans in many industrialized countries (Pavio et al., 2015). Domestic pig (*Sus scrofa domesticus*) and wild boar (*Sus scrofa*) are considered the main animal reservoirs for HEV‐3 and HEV‐4 (Caruso et al., 2017; Johne et al., 2014). A distinct subtype of HEV‐3 has been repeatedly detected in rabbits (*Oryctolagus cuniculus*) (Spahr et al., 2017) and was recently also identified in a few human patients in France (clade HEV3‐ra). However, the route of infection could not be identified as none of the patients had direct contact with rabbits. The authors suggested that foodborne or waterborne infections may take place (Abravanel et al., 2017).

Transmission of HEV‐3 and HEV‐4 by human–animal direct contact has been described. Persons with professional exposure to domestic pigs and wild boars such as slaughterers, pig farmers, forestry workers, hunters or veterinarians exhibit significant higher anti‐HEV antibody prevalences than the general population (Dremsek et al., 2012; Meng et al., 2002; Pavio et al., 2015; Schielke et al., 2015). Moreover, foodborne infections with HEV‐3 and HEV‐4 due to consumption of undercooked meat and meat products derived from infected pigs, wild boars and deer have been repeatedly reported. HEV can be transmitted by milk consumption while other types of food like berries and shellfish have been suspected to act as means for HEV transmission after contamination with animal faeces (Pavio et al., 2015; Spahr et al., 2017). Transmission of HEV‐3 has also been described by parenteral routes due to blood tranfusion or organ transplantation (Abravanel et al., 2017; Kamar, Rostaing, & Izopet, 2013; Kamar et al., 2008).

Several studies conducted in European and Asian countries revealed the presence of HEV antibodies or HEV RNA in domestic and wild animals including domestic pig (*Sus scrofa domestica*), wild boar (*Sus scrofa*), red deer (*Cervus elaphus*), fallow deer (*Dama dama*), roe deer (*Capreolus capreolus*), sika deer (*Cervus nippon*) and wild rabbit (*Oryctolagus cuniculus*) (Spahr et al., 2017). A recent study showed that 52.6% of the *Lepus africana* examined in the region of Burcina Faso, West Africa, was seropositive for HEV (Ouoba et al., 2019). Concerning the EBH, a seroprevalence of 2.2% was reported in in Germany while HEV RNA was not detected in any of the samples examined. In the same study, a seroprevalence of 37.3% was observed in wild rabbits and 17.1% of the samples were HEV RNA positive. This low seroprevalence in hares is indicative of their assumed minor importance in the epidemiology of HEV in Germany while the reported difference between the two species may be attributed to their different habitats and biological behaviours; European brown hares live in open fields, whereas wild rabbits live in their own burrows with increased risk for pathogens transfer to each other due to closer social contacts (Hammerschmidt et al., 2017). Previous studies in European brown hare in Italy provided no evidence of antibodies against HEV (Mazzei & Forzan, 2015) or HEV RNA in hares (Serracca et al., 2015).

## 3 BACTERIAL PATHOGENS

### 3.1 *Yersinia* spp.

*Yersinia pseudotuberculosis* has a worldwide distribution in humans and wild and domestic animals. It has been isolated from carnivorous, herbivorous and omnivorous animals and it is carried subclinically by a range of animal species, including wild mammals, birds and rodents (Najdenski & Speck, 2012). Despite its importance in human and animal health as a pathogen and zoonotic agent, the epidemiological features of yersiniosis are still unclear. The principal reservoirs of *Y. pseudotuberculosis* are rodents and birds while hares, common voles (*Microtus arvalis*) and water voles (*Arvicola terrestris*) are also known to serve as reservoirs (Najdenski & Speck, 2012). Recent surveys suggest that pigs are the primary reservoir of human pathogenic *Yersinia enterocolitica* and, to a lesser extent, of *Y. pseudotuberculosis* in Europe (Bonardi et al., 2016; Vanantwerpen, Damme, Zutter, & Houf, 2014). Although most of the susceptible mammalian species may be subclinical carriers of *Y. pseudotuberculosis*, under certain conditions of stress such as in winter months, when animals, particularly free‐living species, are exposed to cold and starvation and in high concentrations of animals, latent infection may manifest as clinical disease (Najdenski & Speck, 2012).

Faecal‐oral transmission is the main route of natural *Y. pseudotuberculosis* infection. Τhe agent is found ubiquitously in the environment where it can survive for a long time. The environment itself is contaminated from the faeces of infected animals, mainly rodents and birds (Najdenski & Speck, 2012). *Y. pseudotuberculosis* has been isolated from fresh water such as river, well and mountain stream (Fukushima, 1992; Fukushima, Gomyoda, Tsubokura, & Aleksić, 1995). Ingestion of environmental contaminated substances at pasture or watering places and preying upon infected animals are considered important routes of transmission (Fratini et al., 2017; Najdenski & Speck, 2012). The contact between livestock and wildlife is the most important factor in disease transmission (Bengis, Kock, & Fischer, 2002). As for alternative routes of transmission, the role of insect vectors needs further elucidation, milk‐borne spread is possible in cases of yersinial mastitis, venereal transmission through semen is possible, transplacental spread to the foetus has been recorded in several species and vertical transmission is also a possibility (Hubbert, 1972; Najdenski & Speck, 2012). Human infection with *Y. pseudotuberculosis* and *Y. enterocolitica* is acquired by direct or indirect contact with domestic animals, wild animals, birds or consumption of contaminated food and water (Najdenski & Speck, 2012).

Pseudotuberculosis is a typical disease of lagomorphs as it is commonly diagnosed in hares and it constitutes one of the most important and frequent causes of death in hare populations with losses of up to 50% (Frandölich et al., 2003; Wobeser, Campbell, Dallaire, & McBurney, 2009). It is estimated that every hare will have been exposed to *Y. pseudotuberculosis* at some point during its life time (Frandölich et al., 2003). The course of disease is acute to chronic with dyspnoea and diarrhoea. Clinical signs include mild‐to‐severe enteritis, enlargement of the spleen and various lymph nodes (Frandölich et al., 2003). Post‐mortem examination of infected subjects reveals granulomatous nodules in several organs, multifocal caeseous necroses in spleen, liver, intestine and mesenteric lymph nodes (Frandölich et al., 2003; Najdenski & Speck, 2012; Wobeser et al., 2009).

A study performed in Germany on 230 European brown hares revealed a seroprevalence of 89.6% for *Y. pseudotuberculosis* and *Y. enterocolitica* (Bartling et al., 2004). In another study, antibodies against *Yersinia* spp. were found in 55% of European brown hares examined. In contrast, studies in other parts of Germany reported seroprevalences ranging from 13% to 17% (Frandölich et al., 2003). It has been proposed that this difference may have been attributed to failure to diagnose *Y. enterocolitica* infected animals due to the use of laboratory techniques based on Lipopolysaccharide (LPS) antigen and a possible increase in *Yersinia* infection in European brown hares (Frandölich et al., 2003). *Y. pseudotuberculosis* was isolated from 13% of hares found dead in Schleswig‐Holstein, Germany, whereas *Y. enterocolitica* was isolated from only 4% of these hares (Wuthe, Aleksić, & Kwapil, 1995). In a study conducted recently in Italy, animal *Y. pseudotuberculosis* isolates were characterized for the O‐genotype and the majority of hare isolates belonged to O:1a and O:1b serotypes which is common for isolates collected from Western European countries. Besides, strains belonging to O:1 serotype are the most common cause of yersiniosis in humans, followed by strains belonging to the O:2 and O:3 serotypes (Chiesa, Pacifico, Nanni, Renzi, & Ravagnan, 1993; Wunderink et al., 2014). However, O:2a serotype was also detected in hares and, interestingly, serotype O:12–O:13 was found in a hare from Northern Italy; this was the first report of this particular serotype in Europe (Magistrali et al., 2015). The introduction of hares from Eastern European countries during repopulation or the introduction of strains by migratory birds was suggested by the authors as possible explanation for this finding, highlighting the introduction of *Y. pseudotuberculosis* strains in Italy from Far Eastern countries in the past (Magistrali et al., 2015).

### 3.2 *Brucella* spp.

Infections by *Brucella* spp. have been found worldwide in a great variety of terrestrial domestic and wildlife species as well as a wide variety of marine mammals (Godfroid, 2012; Seleem, Boyle, & Sriranganathan, 2010).

The consumption of aborted faetuses and faetal membranes or contaminated food is an important route of transmission in animals. However, the bacteria may also spread through venereal, conjunctival–mucosal and transplacental routes (EFSA, 2009). Regarding *Brucella* spp. infection in humans, in brucellosis endemic regions, infection may be acquired via contact with infected animals or consumption of their products, mostly milk and milk products (e.g. cheese made from unpasteurized milk of sheep and goats and rennet from infected lambs and kids). Farm workers, veterinarians, ranchers and meat‐packing employees are considered at higher risk and brucellosis is referred as an occupational disease for these groups (Tabak et al., 2008). Person‐to‐person transmission occurs rarely. Consequently, as it has been shown previously, eradication of the disease from the natural animal reservoirs leads to a dramatic decrease in the incidence of human infection (Cook et al., 2002; Seleem et al., 2010).

*Brucella* spp. infection in wild animals is not always the result of pathogen spillover from domestic animals to wildlife, but it may represent a sustainable infection in wild animals (Godfroid, 2012). The presence of *Brucella* spp. carriers among wild animals is an established fact; hares, wild reindeer, bison and some rodent species carry *Brucella* spp. regardless of their prevalence among the main hosts (Zheludkov & Tsirelson, 2010). As for hares, this species has been shown to be infected by *B. suis* biovars 1 and 2 (Fort et al., 2012) and along with wild boars (*Sus scrofa*), hares are an important reservoir of *B. suis* biovar 2 (Godfroid, 2012). Biovar 1 is highly pathogenic and cause severe disease in humans while biovar 2 is rarely pathogenic or non‐pathogenic to humans and has only exceptionally been described as the causative agent of human brucellosis (Paton, Tee, Vu, & Teo, 2001; Teyssou et al., 1989). However, the importance of *B. suis* biovar 2 stems for the fact that it can infect domestic pigs and even cattle (EFSA, 2009). Thus, hares and wild boars may serve as a source of infection for free ranging pigs and cattle due to cross‐border and long‐distance movements through migration of wild boars or live animal translocations of hares (Godfroid, 2012; Kreizinger et al., 2014). Moreover, dead hares can apparently be the source of infection for wild and domestic animals and birds of prey (Zheludkov & Tsirelson, 2010).

The role of hares in the dissemination of *B. suis* is probably of lesser importance than that played by wild boar. Data on the geographical distribution of *B. suis* infection in European brown hare, although limited compared with wild boar, are suggestive of a less important prevalence in this species. The species ecology, that is a non‐migratory way of life and occupation of small home ranges, most probably leads to a patchy pattern of the foci of infections and to a reduced interface with other wildlife (Godfroid, Garin‐Bastuji, Saegerman, & Blasco, 2013). Occurrence of *B. suis* in free‐ranging hares reflects their previous or current direct contact with infected wild boars or domestic pigs as well as contact by indirect ways in the same ecological niche where wild boars and hares are present (EFSA, 2009; Frandölich et al., 2003). Thereafter, hares can maintain *B. suis* biovar 2 and infect domestic animals (grazing pigs and cows) even in the absence of a wild boar population (Godfroid et al., 2005). In fact, between 1929 and 1999, ten clinical outbreaks of *B. suis* biovar 2 in domestic pigs in Denmark were linked to hares (Godfroid et al., 2013). During five *Brucella* spp. epizootics in pigs in Denmark, approximately 4,500 hares were examined, and around 3% were *B. suis* positive. Interestingly, the areas of epizootics among domestic pigs coincided with the foci of hare brucellosis (Zheludkov & Tsirelson, 2010). Hares were also considered as a possible source of *B. suis* biovar 2 outbreaks in domestic pigs via swill feeding with offal from hunted infected hares. This species has been deemed as the source of *B. suis* biovar 2 infections in cattle in Denmark, as there is no established population of free‐ranging wild boar in the area (Godfroid et al., 2013). A study conducted recently, showed that European *B. suis* strains of hare origin were closely related to one another and they did not cluster according to their geographic origin. The results of this study were suggestive of cross‐border live animal translocation and of certain *B. suis* strains adaptation to hares. European isolates from domestic pigs were closely related to isolates of hare and wild boar origin, indicating that these species are a source of brucellosis in domestic pigs (Kreizinger et al., 2014). Hares and rabbits are relatively resistant to *B. melitensis* infection. Therefore, it seems more probable that farm animals acquire infection from hares than the opposite. Such cases were reported from Latvia where two *B. abortus* strains were isolated from 10 dead hares with post‐mortem findings indicative of brucellosis (Zheludkov & Tsirelson, 2010).

Reported seroprevalences in hares range from 0% to 17% (Winkelmayer, Vodnansky, Paulsen, Gansterer, & Treml, 2005) (Table 1). Ιn Lower Austria *Brucella* spp. were detected in 2.7% of hares, with higher rates in the north‐eastern districts and in Styria, *Brucella* spp. was isolated from 4.5% of European hares (Winkelmayer et al., 2005).

**Table 1:** Selected studies on the prevalence of antibodies against *Brucella* spp. in *Lepus europaeus* and the serological methods applied

Country	Number of samples examined	Method	Percentage	References
Schleswig‐Holstein, Germany	321	Rose Bengal	0%	Frandölich et al., 2003
Austrian – Czech border region	384	Slow agglutination	3.54% (Austria)	Winkelmayer et al., 2005
			0% (Chech)	
South Moravia, Czech Republic	1,051	Slow agglutination	1.6%	Treml et al., 2007
Hungary	510	Rose Bengal	5/510	Gyuranecz, Erdélyi, et al., 2011

The infection in hares is either latent or it is characterized by inflammation and abscesses in the reproductive system or abscesses in the lymph nodes, liver, spleen, kidneys, urinary bladder, joints and brain (Godfroid et al., 2005; Gyuranecz, Erdélyi, et al., 2011; Kreizinger et al., 2014). Brucellosis was reported to be more prevalent in adult hares compared with subadult ones which may be attributed to its chronic nature and the spread during reproduction (Treml et al., 2007). In general, it seems that this pathogen contributes only little to the overall mortality of hares (Winkelmayer et al., 2005).

Natural foci of brucellosis seem to be independent of the hare population density which can be hypothetically explained by the so‐called small home range and a sufficiently low threshold of the disease in this species (Kunst, Wal, & Wieren, 2001; Pikula, Beklova, Holesovska, Skocovska, & Treml, 2005). Other factors such as climatic factors that influence the survival of the pathogen in the environment and the chronicity of the disease may affect the maintenance of natural foci of brucellosis (Pikula et al., 2005). The disease may exist in the hare environment thus constituting a threat of contracting the pathogen, independently of the hare population density or the territory is brucellosis free. In the latter case, animal translocation due to re‐populating efforts in hunting grounds or migration of the wild boar can bring *Brucella* spp. in a brucellosis‐free territories (Pikula et al., 2005).

### 3.3 Francisella tularensis

*F. tularensis* has a remarkably broad host range and complex epidemiology. Currently, four *F. tularensis* subspecies have been recognized; *tularensis* (type A strains), *holarctica* (type B strains), *mediasiatica* and *novicida*. Worldwide, tularemia is caused by the subspecies *tularensis* and *holarctica*. Type A strains are mainly found in North America, but a few have been isolated in Slovakia and Austria. They are the most virulent strains, being responsible for 90% of human cases, while lagomorphs are the reservoir species. Only type B strains have been reported to cause tularemia in Europe. Type B strains are reported throughout the northern hemisphere, but isolates have also been identified in Tasmania. *F. tularensis* subspecies *holarctica* strains are typically separated into three biovars; Biovar 1 (erythromycin sensitive) reported in western Europe, biovar 2 (erythromycin resistant) found in eastern European countries with some overlapping in their geographical spread and biovar japonica (ferment glycerol) mainly found in Japan, but being also reported in China and Turkey (Dumas, 2005; Maurin & Gyuranecz, 2016).

Natural *F. tularensis* infection has been reported in a wide range of vertebrates, including mammals, birds, amphibians and fish, and in certain invertebrates (Gyuranecz, 2012). The disease primarily concerns the genera *Lagomorpha* and *Rodentia*. The European brown hare (*Lepus europaeus*) is a common host of *F. tularensis* in Central Europe and this species is moderately sensitive to *F. tularensis* infection. On the other hand, the disease occurs frequently in mountain hares (*Lepus timidus*) in Scandinavia and Russia and it is often fatal. Thus, European brown hare is regarded as a reservoir due to its possible capability to maintain the pathogen for a longer period than the mountain hare (Gyuranecz et al., 2010). Rodents play an important role in the maintenance of enzootic foci in Eurasia while voles are most frequently involved in tularaemia epizootics. In fact, the water vole (*Arvicola amphibius*) and the common vole (*Microtus arvalis*) are highly susceptible to the disease and they may also present chronic infection, thereby acting as reservoirs the periods in between the epizootics (Gyuranecz, 2012). However, as small rodents and lagomorphs often develop fatal infections, the existence of alternative unidentified reservoirs such as domestic animals, able to transmit the disease to humans, is also possible (Weinberg & Branda, 2010). Besides, although infection is rare among domestic animals, outbreaks can occasionally occur among sheep during lambing season (Gyuranecz, Rigó, et al., 2011). Haematophagous arthropods, especially ticks, serve as mechanical as well as biological vectors; they enable amplification of the bacteria thus contributing to re‐transmission, and they maintain the bacterium throughout its multiple life stages (Gyuranecz, 2012).

The two known transmission cycles of tularaemia are the terrestrial and the aquatic cycle. In the terrestrial cycle, hares and rodents are the most important mammalian hosts contaminating the environment through their body discharges, whereas haematophagous arthropods serve as vectors (Gyuranecz, 2012). Other routes of transmission that have been described in hares in Europe include aerogenous infection (Gyuranecz et al., 2010) and the alimentary route in mountain hares in Scandinavia (Mörner, Sandström, Mattsson, & Nilsson, 1988). In the aquatic cycle, voles and possibly muskrats and beavers serve as the main host species. Water contamination could be maintained by the faecal matter of infected animals, or by infected animal carcasses (Gyuranecz, 2012).

The major modes of *F. tularensis* transmission to humans include direct transmission from the animal reservoir, arthropod bites and transmission through contaminated water and soil. Human tularaemia cases from aquatic sources are more common and often occur as large outbreaks (Maurin & Gyuranecz, 2016). Direct transmission through handling the meat and fur of an infected animal, mainly a brown hare, might also occur as the bacterium is capable of penetrating healthy skin (Richard & Oppliger, 2015). This is the main mode of human infection in Central Europe (Keim, Johansson, & Wagner, 2007; Richard & Oppliger, 2015). Ingestion of undercooked meat prepared from an infected animal, or animal bites (especially from small rodents, cats and dogs) represents an additional danger (Keim et al., 2007; Richard & Oppliger, 2015). Arthropod‐borne transmission is common in the United States after a tick bite, a mosquito bite or, rarely, bites from other arthropods (Richard & Oppliger, 2015). *F. tularensis* can persist for weeks to months in the environment. Thus, inhalation of contaminated dusts aerosolized from soil, faecal matter and dead animals, contact or ingestion of contaminated water (e.g. drinking water from tanks and wells) are other routes of transmission (Maurin & Gyuranecz, 2016; Richard & Oppliger, 2015).

In a study conducted recently in Hungary, the factors influencing the emergence of tularaemia were investigated and the authors suggested that the number of tularaemia cases in humans was positively associated with the seroprevalence of *F. tularensis* among European brown hares and the population density of common voles (Gyuranecz et al., 2012). The high vole population density leads to increased transmission and spillover to hares by stress‐related aggression, cannibalism and contamination of the environment by infectious body discharges. This enhanced transmission and spillover may lead to epizootics and the spread of the disease may be further facilitated by the bacterial shedding in the urine of the infected hares (Gyuranecz et al., 2012, 2010). It becomes clear that the presence of a high number of sources of infection may lead to increased human tularaemia cases via the handling and skinning of hares, and, potentially, the inhalation of infectious aerosols. Moreover, as rodents and lagomorphs are hosts of blood sucking arthropods, a high number of infected hosts may lead to an increased number of infected ticks and thereby, increased transmission of the pathogen to humans. Interestingly, the hare population density was negatively correlated to the seroprevalence of *F. tularensis* in hares (Gyuranecz et al., 2012, 2010).

Although the EBH is considered a moderately susceptible species, some authors have suggested that this species is highly susceptible to *F. tularensis.* In fact, a heterogeneous response to infection has been described in hares; some die of overwhelming bacteraemia and others survive with a protracted course of infection (Gyuranecz et al., 2012). Marked bacteraemia may be caused by even extremely low infectious doses of the bacterium and the infected hares may be an important source of infection for blood sucking arthropods and their carcasses and excrements may contaminate the environment. A chronic form of the disease has been described for those individuals that survive infection being a permanent source of infection for other animals sharing the same living space as well as for humans (Treml et al., 2007). Other studies have suggested that hares, along with infected ticks, may serve as *F. tularensis* reservoirs between epizootics (Gyuranecz et al., 2012). Hares seroconvert, and potentially they can carry viable bacteria over a longer time span and thus serve as a reservoir species (Gyuranecz et al., 2010).

In moderately susceptible species such as the European brown hare, the disease can be subacute. In the late stages of the disease, the clinical manifestations include depression, stupor, loss of body weight and lack of fear, facilitating capture. At necropsy, numerous, randomly distributed, well‐demarcated, greyish‐white or yellowish‐white foci can be observed most frequently in the lungs, the pericardia and the kidneys and sometimes in the testicles, bone marrow and mammary glands (Gyuranecz et al., 2010). A recent study revealed that subpopulations of *F. tularensis* subsp. *holarctica* (clade B.13 and B.FTNF002‐00) may be associated with different pathologic findings in the European brown hare. In fact, in natural infection with *F. tularensis subsp. holarctica* (clade B.13), the most commonly affected tissues are lung, pericardium and kidney (Gyuranecz et al., 2010). On the other hand, strains of the clade B.FTNF002‐00 were almost invariably associated with splenitis and hepatitis (Origgi & Pilo, 2016).

Previous studies in other European countries showed that the seroprevalence of *F. tularensis* in hares range from 0% (Frandölich et al., 2003) to 7% (Winkelmayer et al., 2005) (Table 2). In Germany, during 2006–2009, the DNA prevalence of *F.tularensis* in European Brown hare was 1.1%. Importantly, the prevalence was higher for the hares found dead compared with the hunted ones (2.9% and 0.7%, respectively) suggesting that hares became seriously diseased following infection and died (Runge et al., 2011).

**Table 2:** Selected studies on the prevalence of antibodies against *F. tularensis* in *Le. europaeus* and the serological methods applied

Country	Number of samples examined	Method	Percentage	Reference
Austria	110	MAT	4.5%	Höflechner‐Pöltl, Hofer, Awad‐Masalmeh, Müller, & Steineck, 2000
Germany	299	Western blotting	0%	Frölich et al., 2003
Austrian – Czech border region	384	Slow agglutination	6%	Winkelmayer et al., 2005
South Moravia, Czech Republic	1,051	Slow agglutination	6.5%	Treml et al., 2007
Hungary	197	Agglutination test	5.1%	Gyuranecz, Erdélyi, et al., 2011

## 4 PROTOZOAL PATHOGENS

### 4.1 Toxoplasma gondii

*Toxoplasma gondii* is a significant zoonotic obligate intracellular parasite with a worldwide distribution that affects humans and warm‐blooded animals. The only known definitive hosts for *T. gondii*, able to produce and shed the environmentally resistant stage, oocysts, in their faeces are the domestic and free‐ranging felids. The parasite reproduces in the small intestine of the felids, and millions of oocysts are shed into the environment (Dubey & Dubey, 2010). The sporulated oocysts are being ingested by intermediate hosts, which are potential prey for felids and which include almost all warm‐blooded animals, including mammals and birds. The parasite multiplies and encysts in many tissues of the intermediate hosts (Dubey & Jones, 2008). Migrating birds may play an important role in the introduction of the parasite in new areas; local carnivores may be subsequently infected by eating infected prey. Besides, birds worldwide have been shown to be susceptible to *T. gondii* infection and migratory birds, such as geese, overwinter in areas where felids are common and where infectious *T. gondii* oocysts are likely to be found in high numbers in the environment. Thus, it is possible for *T. gondii* to be transmitted from one intermediate host to another (e.g. bird to carnivore) without the need of sexual reproduction of the parasite in a felid definitive host (Gotteland et al., 2014; Jokelainen, 2012; Prestrud et al., 2007; Reiling & Dixon, 2019). Climatological conditions such as humidity, rainfall and temperature impact the oocyst survival and sporulation in localized areas. In fact, a positive and significant correlation of antibody prevalence of *T. gondii* between locations and rainfall has been observed in wildlife species in Spain (Gamarra et al., 2008).

Humans become infected post‐natally by ingesting tissue cysts from undercooked meat, consuming food or drink water contaminated with oocysts, or by accidentally ingesting oocysts from the environment. Consumption of raw or almost raw, dried, cured or smoked meat from domestic animals, unpasteurized goat milk or consumption of meat from wild animals may be associated with ingestion of the parasite (Bartova & Sedlak, 2012). Humans, especially hunters, may also get infected through contact with the parasite while dressing game (Dubey & Dubey, 2010; Fernández‐Aguilar et al., 2013). Frequent contact with animals and soil, such as in the case of abattoir workers, garbage handlers and waste pickers, was recognized as a factor associated with higher *T. gondii* prevalence (Dubey & Beattie, 1988). Moreover, infection by direct contact was reported for children playing with dogs and cats as animals can act as mechanical vectors (Etheredge, Michael, Muehlenbein, & Frenkel, 2004). However, only a small percentage of exposed adult humans develop clinical signs of disease and whether the severity of toxoplasmosis in immunocompetent hosts is due to the parasite strain, host variability or other factors is still under investigation. In fact, attention has been focused on genetic variability among *T. gondii* isolates from apparently healthy and sick hosts (Dubey & Jones, 2008).

*T. gondii* can cause disease in various hosts and it is a frequent cause of early embryonic death and resorption, faetal death and mummification, abortion, still birth and neonatal death in animals. (Bartova & Sedlak, 2012). Regarding hares, they are considered as exceptionally susceptible to primary infection (Gustafsson, Uggla, & Järplid, 1997; Lindsay & Dubey, 2014). Fatal toxoplasmosis has been repeatedly described in hares (Christiansen & Siim, 1951; Gustafsson et al., 1997) and it is frequently found in hare populations (Frölich et al., 2003). Typically, toxoplasmosis in hares is acute and they are in a normal nutritional state (Christiansen & Siim, 1951; Gustafsson, Uggla, Svensson, & Sjöland, 1988). Interstitial pneumonia, multifocal areas of hepatocellular necrosis, spleen enlargement, encephalitis and moderate necrosis of lymphoid follicles of the lymph nodes are the predominant lesions in hares in endemic areas (Christiansen & Siim, 1951; Gustafsson, 1997; Gustafsson et al., 1997).

It has been suggested that stress or environmental factors like winter and harsh climatologic conditions may trigger acute fatal toxoplasmosis in hares (Sedlák, Literák, Faldyna, Toman, & Benák, 2000). Besides, most of the fatal toxoplasmosis cases have been reported from Northern countries of Europe, where harsh winter conditions are common. For instance, in a study conducted previously in the Czech Republic, all the hares that died during the experiment were kept in outdoor boxes in temperatures below 0°C (Sedlák et al., 2000). In Denmark and Sweden, most of the fatal cases were observed in animals sampled during the cold months. However, an outbreak in Japan occurred during the warm season (Christiansen & Siim, 1951; Shimizu, 1958). Hares get infected through ingestion of food or water contaminated with oocysts from domestic cat or free‐ranging felid faeces with which they share the same habitats (Lindsay & Dubey, 2014). Infected hares can act as a potential source of *T. gondii* for other animals, especially for carnivores but also for humans (Bártová et al., 2010).

Experimental infections have demonstrated that toxoplasmosis in hares differs from that in rabbits (*Oryctolagus cuniculus*). In a previous study, post‐mortem examination 1 week after inoculation with 50 oocysts of a local *T. gondii* isolate in mountain hares revealed the presence of parasites in the majority of the tissues examined and extensive damage. Only minor lesions were observed in rabbits. No differences in the production of antibodies were observed, but the mountain hares seemed to lack a proper cellular response (Gustafsson, 1997; Gustafsson & Uggla, 1994). In another study, 12 brown hares were inoculated with 10, 1,000 or 10,000 oocysts and all of them died, whereas, only 2 of the 12 rabbits, suffering from a concurrent accidental *Pasteurella* infection, finally died (Sedlák et al., 2000). *T. gondii* was isolated from liver, brain, spleen, kidney, lung, heart and skeletal muscles (Sedlák et al., 2000).

The proposed natural, inherent susceptibility of hares to *T. gondii* is well supported by cases of fatal toxoplasmosis, with a concomitant low prevalence of latent chronic or subclinical infections (Christiansen & Siim, 1951; Jokelainen, Isomursu, Näreaho, & Oksanen, 2011). In contrast, there are other studies that reported high *T. gondii* seroprevalence in European brown hares (Frölich et al., 2003), suggesting they can survive *T. gondii* infection in the wild. The explanations for the failure to achieve equilibrium between the host and the parasite are controversial and focus on the characteristics of the host (Gustafsson et al., 1997; Maubon, Ajzenberg, Brenier‐Pinchart, Dardé, & Pelloux, 2008; Sedlák et al., 2000).

*T. gondii* antibodies have been found in animals worldwide and although for most host species, the seroprevalence numbers are clearly higher than incidence of clinical and fatal cases, European brown hare appears exceptionally susceptible to the infection (Jokelainen et al., 2011). Seroprevalence of *T. gondii* in hares varies among countries ranging from 0% to 46% (Table 3). Although different serological techniques were used, the differences in the serological status of hare population against *T. gondii* across European countries are indeed highlighted. Besides, *T. gondii* seroprevalence varies even within different areas of a country and within the same city (Jokelainen et al., 2011). In a previous study in Spain, significantly higher seroprevalence was observed in juvenile Iberian hares compared with the adult ones (Fernández‐Aguilar et al., 2013). The authors suggested that possibly *T. gondii* infection affects the survival of infected hares and thus infected juveniles reach the adult stage at lower rates than the non‐infected hares or that a short‐lived humoral immune response against *T. gondii* exists in Iberian hares (Fernández‐Aguilar et al., 2013). Nevertheless, the dynamics of *T. gondii* antibodies in hares is not well understood as it has only been analysed in short‐term experimental infections because of hares suffering or dying following infection (Gustafsson, 1997; Sedlák et al., 2000).

**Table 3:** Selected studies on the prevalence of antibodies against *T .gondii* in *Le. europaeus* and the serological methods applied

Country	Number of samples examined	Method	Percentage	References
Sweden	176	IFAT, ELISA, DAT, Sabin‐Feldman test	0%	Gustafsson & Uggla, 1994
Schleswig‐Holstein, Germany	318	Sabin‐Feldman test	46%	Frandölich et al., 2003
France	23	MAT	9%	Aubert et al., 2010
Czech Republic	333	IFAT	21%	Bartova et al., 2010
Slovakia	209		6%	
Austria	383		13%	
Italy	222	MAT		Ebani et al., 2016
Greece	105	IFAT	5.7%	Tsokana et al., 2019

Regarding fatal toxoplasmosis cases in hares, in Sweden, toxoplasmosis was the cause of death in 10% of European brown hares and 4% of mountain hares necropsied in the 1980s (Gustafsson et al., 1988). In Denmark a similar proportional mortality rate of almost 10% in brown hares was also reported earlier (Christiansen & Siim, 1951). In Germany, 57% of the hares examined were immunohistochemically positive for *T. gondii* antigen (Frölich et al., 2003). In addition to these findings from wild hares, an outbreak of acute toxoplasmosis with high mortality was described on a mountain hare ranch in Japan (Shimizu, 1958).

### 4.2 *Leishmania* spp.

Protozoan parasites of the genus *Leishmania* (*Trypanosomatida*: *Trypanosomatidae*) are the causative agents of a complex of vector‐borne diseases, Leishmaniases, which represent an important public health concern. Approximately 53 *Leishmania* species have been described; of these, 31 species are known mammal parasites and 20 species are pathogenic for humans. They are present in extremely diverse ecosystems and are able to infect a wide range of mammals (Bañuls, Hide, & Prugnolle, 2007).

The transmission mainly occurs through the bites of infected female sandflies of the genus *Phlebotomus* in the Old World and *Lutzomyia* in the New World. Approximately 166 sandfly species have been reported to be proven or potential vectors of different *Leishmania* species in the Old and New World. Among these species, 78 are reported as the proven vectors of *Leishmania*. Among the above‐mentioned sandfly vectors, 7 are involved in the transmission of *L. major*, 7 in the transmission of *L. tropica*, 31 in the transmission of *L. infantum* and 9 in the transmission of *L. donovani* (Akhoundi et al., 2016). *Leishmania* species are heteroxenous as they infect the phagocytes of the reticuloendothelial system of mammals and the intestinal tract of phlebotomine sandflies. However, other arthropods such as *Forcipomyia* spp. (Diptera: *Ceratopogonidae*) as well as some tick species have been reported as the potential vectors of *Leishmania* spp. (Dantas‐Torres et al., 2010; Slama et al., 2014; Solano‐Gallego et al., 2012). Importantly, sexual, vertical and iatrogenic transmission have been also reported (Morillas‐Marquez et al., 2002; Pangrazio et al., 2009; Silva et al., 2009).

The successful zoonotic or anthroponotic transmission between the sandfly vector and a mammalian reservoir is a key factor for survival of the *Leishmania* parasite. Zoonotic transmission cycles involve a reservoir host such as dogs in domestic cycles and rodents, marsupials, edentates, monkeys and wild canids in sylvatic cycles where enzootic transmission occurs between wild animals, with humans forming dead‐end hosts if infected. It is worth mentioning that sandflies use wild animal burrows as breeding sites (Feliciangeli, 2004). Thus, it has been suggested that in the case of *Phlebotomus perniciosus* a possible overlapping of wild and domestic environments may occur due to the widespread presence of this sandfly species in both environments (Millán, Ferroglio, & Solano‐Gallego, 2014). In the strictly anthroponotic transmission cycles humans with kala‐azar, post–kala‐azar dermal leishmaniasis (PKDL) and to a lesser extent those with asymptomatic infection are the sole reservoirs (Bern, Maguire, & Alvar, 2008; Quinnell & Courtenay, 2009). The specific *Leishmania* spp. are transmitted either anthroponotically or zoonotically. However, exceptions such as *Leishmania tropica* which although having a predominant anthroponotic cycle, may be transmitted from animals, also exist (Pace, 2014).

*Leishmania* infection has been documented in various wild mammalian species, including carnivores (Beck et al., 2008; Luppi et al., 2008), primates (Malta et al., 2010), marsupials (Santiago et al., 2007), edentates (De Araújo, Boité, Cupolillo, Jansen, & Roque, 2013), lagomorphs (Molina et al., 2012; Ruiz‐Fons, Ferroglio, & Gortázar, 2013a; Tsokana et al., 2015), bats (De Lima et al., 2008) and rodents (Kassahun et al., 2015; Papadogiannakis et al., 2010). However, a fundamental factor determining the ability of a species to act as a potential reservoir is its infectiousness to sandflies. In Europe, this capacity has been demonstrated for the black rat (*Rattus rattus*) in Italy (Gradoni, Pozio, Gramiccia, Maroli, & Bettini, 1983) and the Iberian hare in Spain (Molina et al., 2012). At late 2010, a surprising outbreak of 446 human leishmaniasis clinical cases was recorded, centred on a suburban park, in Fuenlabrada, south‐western Madrid (Spain). However, the unchanged incidence in dogs in the same period, prompted the researchers to investigate the role of the Iberian hare in the epidemiology of *L. infantum*. According to different authors, in that municipality, 30% to 45% of hares were *Leishmania* infected (Arce et al., 2013; Moreno et al., 2014). The molecular typing of hare isolates showed that the isolates involved in the outbreak belonged to the ITS LOMBARDI subtype of *L. infantum*, as those isolated from humans in different parts of Madrid since at least 1992 (Chicharro et al., 2013). Xenodiagnosis showed that seven apparently healthy naturally infected hares were infectious to a mean 4.7% (0%–10.6%) *Phlebotomus perniciosus*, a competent vector for *L. infantum* suggesting that hares may represent at least a secondary reservoir in the sylvatic transmission cycle of *L.infantum* in that region (Molina et al., 2012)*.* In another study, analysis of blood meals of ten sandflies captured in the affected area showed a high preference for hares (*n* = 6), followed by humans (*n* = 3) and cats (*n* = 1) (Jiménez et al., 2013). Following studies in Spain showed that *L. granatensis, L. europaeus, L. castroviejoi* from six different regions, presented an overall 43.6% DNA prevalence (*Le. granatensis* 42.1%, *Le. europaeus* 56.3%, *L. castroviejoi* 0%) (Ruiz‐Fons et al., 2013a). Later, *L. infantum* infection in hares was found to be 23.49% in Northern Greece (Tsokana et al., 2015), 18.52% in Italy (Zanet et al., 2016) and more recently 1.9% in central Italy (Rocchigiani et al., 2018). A seroprevalence of 74.1% was reported in *Le. granatensis* in Spain (Moreno et al., 2014), whereas in Italy the seroprevalence in *Le. europaeus* was 0.9% (Ebani et al., 2016). Clinical illness has not been detected in seropositive hares (Moreno et al., 2014). It has been suggested that possibly, under specific circumstances such as an unusually high concentration of hares, high density of sandflies and a low level of immunity in the human population (Carrillo, Moreno, & Cruz, 2013) this species can act as reservoir for *L. infantum* infection (Millán et al., 2014).

## 5 CONCLUSIONS

The contribution of hares in the epidemiology of selected emerging zoonotic pathogens is herein discussed; maintenance of high endemicity levels in the case of CCHFV, reservoir of pathogens such as *Yersinia* spp., *B. suis* biovar 2, *F. tularensis* and *L. infantum* and potential source of *T. gondii* for other animals, especially for carnivores but also for humans. However, European Brown hare may also be a host of minor importance as in the case of HEV. While keeping its so‐called “home range”, this species is exposed to many infectious agents of natural nidality (Treml et al., 2007) and shares common pathogens with other wild and domestic animals. Taking into consideration the possible live animal translocations of infected hares and that this species acts as a host of vectors (fleas, ticks, mosquitoes and sandflies) and the prey of carnivores and omnivores that travel in great distances getting into contact with domestic animals and humans, the continuous surveillance of hare populations becomes of great value for the collection of information on the population health status and the pathogens currently circulating in the area posing risk for wildlife, domestic animals and humans. Besides, the hunter‐harvested EBH population is an excellent indicator for recent pathogen transmission due to its short lifespan.

## CONFLICT OF INTEREST

The authors declare that they have no conflict of interest.
